# Natural infections of *Pintomyia verrucarum* and *Pintomyia maranonensis* by *Leishmania (Viannia) peruviana* in the Eastern Andes of northern Peru

**DOI:** 10.1371/journal.pntd.0009352

**Published:** 2021-04-15

**Authors:** Hirotomo Kato, Chisato Seki, Makoto Kubo, Lizandro Gonzales-Cornejo, Abraham G. Caceres

**Affiliations:** 1 Division of Medical Zoology, Department of Infection and Immunity, Jichi Medical University, Tochigi, Japan; 2 Division of Immunology, Kitasato University School of Allied Health Sciences, Kanagawa, Japan; 3 Laboratorio Referencial de Salud Pública and Laboratorio de Entomología, Dirección Regional de Salud Amazonas, Peru; 4 Sección de Entomología, Instituto de Medicina Tropical “Daniel A. Carrión” y Departamento Académico de Microbiología Médica, Facultad de Medicina Humana, Universidad Nacional Mayor de San Marcos, Lima, Peru; 5 Laboratorio de Entomología, Instituto Nacional de Salud, Lima, Peru; National Institute of Allergy and Infectious Diseases, UNITED STATES

## Abstract

The natural infection of sand flies by *Leishmania* was investigated in Andean areas located between the Central and Eastern Cordilleras of northern Peru where cutaneous leishmaniasis caused by *Leishmania (Viannia) peruviana* is endemic. Sand flies were captured at five locations along the Utcubamba River in the Department of Amazonas, and morphologically identified under a microscope. Among 422 female sand flies dissected, the most dominant species was *Pintomyia verrucarum* (320 flies), followed by *Pi*. *maranonensis* (83 flies), *Pi*. *robusta* (13 flies), and *Lutzomyia castanea* (6 flies). Genetic analysis of sand flies from these areas together with those from other areas revealed that individuals of *Pi*. *verrucarum* were closely related regardless of morphological variation of their spermathecae. On the other hand, individuals of *Pi*. *maranonensis* collected in the study area were distant from those of other areas with genetic distances over the intraspecific level but mostly below the interspecific level, suggesting the unique characteristics of sand flies in this area. The natural infection of sand flies by flagellate parasites was detected mainly in the hindgut of each one of *Pi*. *verrucarum* and *Pi*. *maranonensis*. Both parasite species were identified as *L*. *(V*.*) peruviana* based on cytochrome *b* and mannose phosphate isomerase gene analyses. In addition, parasite species obtained from the lesion of a patient with cutaneous leishmaniasis in the study area in this period was identified as *L*. *(V*.*) peruviana*. These results strongly suggest that *Pi*. *verrucarum* and *Pi*. *maranonensis* are responsible for the transmission of *L*. *(V*.*) peruviana* in these areas. This is the first report of the natural infection of *Pi*. *maranonensis by L*. *(V*.*) peruviana*.

## Author summary

Phlebotomine sand flies are tiny insects of the family Psychodidae in the order Diptera, and female sand flies suck blood for egg production. Approximately 1,020 sand fly species have been recorded in the world, of which about 550 species are in the New World. Only a part of them are associated with medically important infectious diseases such as leishmaniasis, and importantly, each vector species transmits specific species of *Leishmania*. Since the infecting *Leishmania* species is the major determinant of the clinical outcome and its endemicity is largely dependent on the prevalence of the vector species, the identification of circulating sand flies and vector species, which determine transmissible parasite species, is important to predict the risk and expansion of the disease in endemic and surrounding areas. However, the vector species involved in disease transmission remains unidentified in most endemic areas because the infection rate in sand fly populations is very low. In the present study, sand flies were investigated in the Department of Amazonas in the Eastern Andes of northern Peru, in which cutaneous leishmaniasis caused by *Leishmania (Viannia) peruviana* is endemic, to clarify the transmission mechanism of leishmaniasis in these areas. In addition, genetic analyses of circulating sand flies were performed to elucidate the characteristics of sand flies in these areas.

## Introduction

Phlebotomine sand flies are blood-sucking insects belonging to the family Psychodidae in the order Diptera [1,2]. To date, 1,020 sand fly species have been recorded in the world, of which about 550 species are in the New World [3]. The identification of sand fly species is medically important since approximately 10 percent of them are responsible for the transmission of human pathogens such as *Leishmania* protozoa [2,4,5]. In addition, each vector species transmits specific *Leishmania* species, and the infecting species is the major determinant of the clinical outcomes, such as cutaneous, mucocutaneous, and visceral disorders [4–6]. Therefore, studies on sand fly fauna and the identification of vector species of leishmaniasis in endemic and surrounding areas are important for predictions of the risk of transmission and expansion of the disease.

Peru is one of the most highly endemic countries for cutaneous leishmaniasis (CL), distributed through the country from highlands to lowlands, whereas mucocutaneous leishmaniasis (MCL) in this country is endemic mostly in Amazonian areas [7,8]. Six *Leishmania* species and several hybrids have been recorded as responsible for leishmaniasis in this country [7–9]. Of these, predominant causative agents are *L*. *(V*.*) peruviana*, *L*. *(V*.*) braziliensis*, and *L*. *(V*.*) guyanensis*, mainly circulating in the Andean highlands, tropical rainforest, and northern to central rainforest areas, respectively [7,8,10,11]. Approximately 190 sand fly species have been recorded in Peru, and information on prevalent sand fly species is accumulating, especially in Andean areas; however, the vector species responsible for the transmission of *Leishmania* remains to be identified in most endemic areas [12]. The vector species of *L*. *(V*.*) peruviana* was identified as *Lutzomyia ayacuchensis* in the western valley of central Andes [13] and *Lu*. *peruensis* in northern and central Andes [14–17]. *Pintomyia verrucarum*, a widely distributing species in Andean highlands, was reported to have the capacity to transmit *L*. *(V*.*) peruviana* under experimental conditions [18], and the natural infection of *Pi*. *verrucarum* by *Leishmania* species was detected by PCR using pooled sand fly samples from the west Andean slope in central Peru, although the parasites were not identified at the species level [19]. In addition, *Lu*. *tejadai* was reported as a vector of a hybrid of *L*. *(V*.*) braziliensis* and *L*. *(V*.*) peruviana* in the central Andes of Peru [20].

In the present study, to further disclose circulating sand fly species and identify the vectors of *Leishmania* protozoa, sand flies were investigated in endemic areas along the Utcubamba River in Provinces of Chachapoyas, Luya, and Bongara, Department of Amazonas located in the Eastern Andes of northern Peru where CL caused by *L*. *(V*.*) peruviana* is endemic [7,8].

## Materials and methods

### Ethics statement

Verbal informed consent was obtained prior to the sample collection, providing information on the process of diagnosis and *Leishmania* species analysis, following the guidelines of the Ethics Committee of the Ministry of Health, Peru. The study was approved by the ethics committee of Jichi Medical University (approval number: 17–080) [8,9].

### Sand fly collection

**Fig 1:**
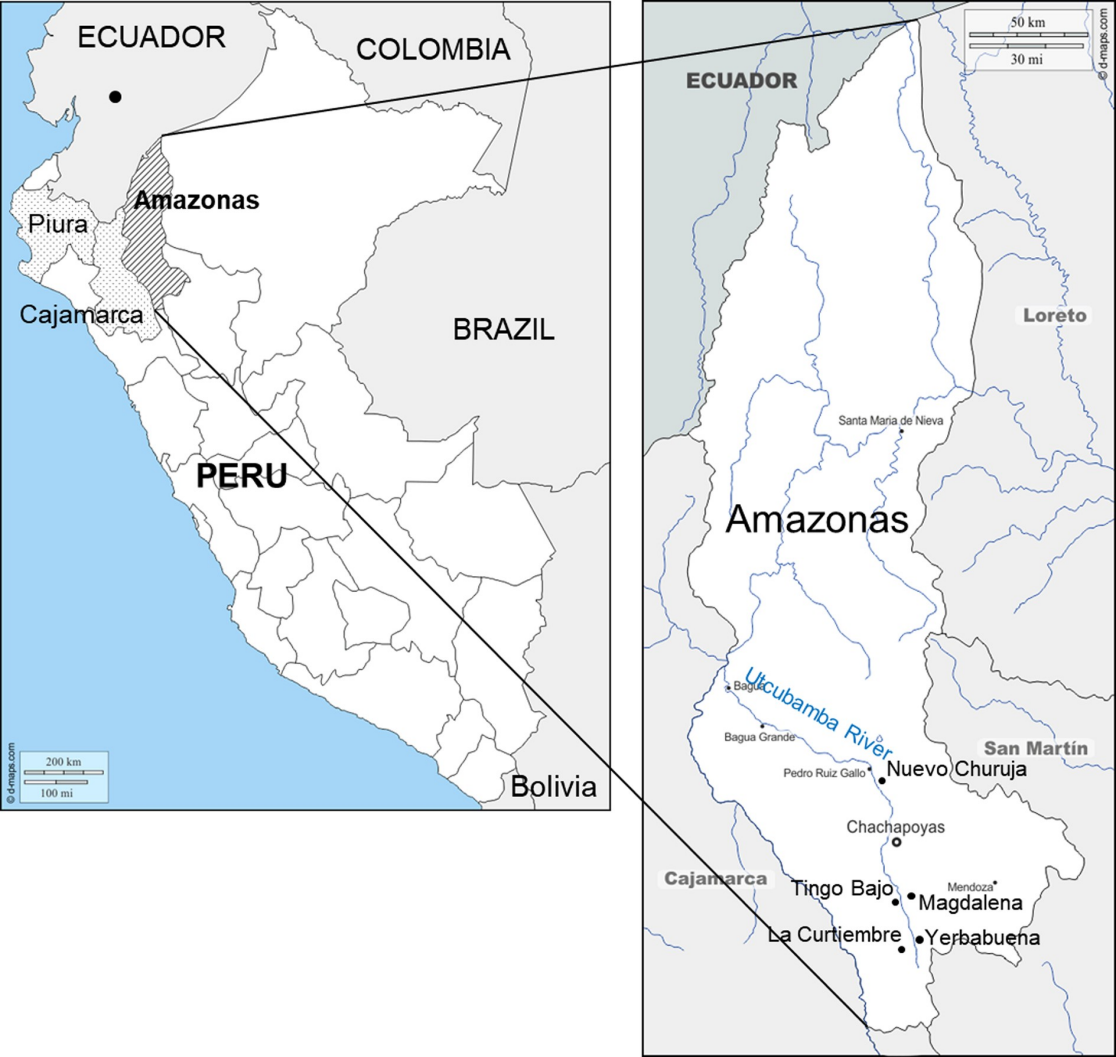
Maps of study areas. Left panel: Department of Amazonas, Peru, is shown by the hatched lines, and Departments of Piura and Cajamarca, Peru, are shown by the small dots. A black spot in Ecuador shows the collection site of *Pi*. *maranonensis* in the Province of Chimborazo, Ecuador. Right panel: Sample collection sites in the Department of Amazonas: La Curtiembre, Yerbabuena, Magdalena, Tingo Bajo, and Nuevo Churuja. (Adapted from a map available at https://www.d-maps.com/carte.php?num_car=4764&lang=en and https://www.d-maps.com/carte.php?num_car=190488&lang=en).

Sand flies were captured with a mouth aspirator on protected human bait between 18:30 and 21:00 and CDC light traps operated throughout the night from 18:00–06:00 for 11 nights in July 2019 around patients’ houses in the rural area at five localities along the Utcubamba River in the Provinces of Chachapoyas, Luya, and Bongara, Department of Amazonas (Fig 1). Female sand flies were dissected and identified at the species level mainly based on the morphology of their spermathecae [6,21]. They were also examined under light microscopy for natural flagellate infections, and samples were fixed individually in absolute ethanol. Ethanol-fixed specimens were dried up and individually lysed in 50 μL of DNA extraction buffer [150 mM NaCl, 10 mM Tris-HCl (pH 8.0), 10 mM EDTA and 0.1% sodium dodecyl sulfate (SDS)] containing proteinase K (100 μg/mL). The samples were incubated at 37°C overnight, heated at 95°C for 5 min, and then 0.5 μL of each sample was directly used as a template for PCR amplification [16,17,20,22,23].

### Clinical samples

A clinical sample was collected from a patient suspected of having CL. A tissue sample was taken by scraping the margins of an active cutaneous lesion of the patient, spotting the scrapings onto an FTA Classic Card (Whatman, Newton Center, MA) and storing it at room temperature. Two-mm-diameter disks containing the sample spot were punched out from the card and washed three times with FTA Purification Reagent (Whatman) and once with Tris-EDTA buffer. The disks were air-dried and directly subjected to PCR amplification [7,8,20].

### Identification of *Leishmania* species

*Leishmania* species were identified by cytochrome *b* (*cyt* b) gene sequence analysis [8,9,16]. PCR amplification with a pair of specific primers: L.cyt-S (5’-GGTGTAGGTTTTAGTYTAGG-3’) and L.cyt-R (5’-CTACAATAAACAAATCATAATATRCAATT-3’), was carried out with 30 cycles of denaturation (95°C, 1 min), annealing (55°C, 1 min), and polymerization (72°C, 1 min) using Ampdirect Plus reagent (Shimadzu Biotech, Tsukuba, Japan). PCR products were directly cloned into the plasmid using a pGEM-T Easy Vector System (Promega, Madison, WI), and the sequence of the insert was determined by the dideoxy chain termination method using a BigDye Terminator v.3.1 Cycle Sequencing Kit (Applied Biosystems, Foster City, CA).

### Differentiation between L. (V.) braziliensis and L. (V.) peruviana

Differentiation between *L*. *(V*.*) braziliensis* and *L*. *(V*.*) peruviana* was performed by PCR-RFLP analysis of the mannose phosphate isomerase (*mpi*) gene, as described previously [8,9,16]. Briefly, the *mpi* gene fragment was amplified with a pair of primers: MPI-S (5’-GCTCTTCCTGTCGGACAGCGAGC-3’) and MPI-R (5’-TCACTCTCGAAGGGAGTTCG-3’), and digested with the restriction enzyme, *Vpa*K11BI (Takara Bio, Shiga, Japan). The RFLP pattern was analyzed by 3% agarose gel electrophoresis.

### Sequence analysis of sand fly cytochrome oxidase I (COI) gene

The sand fly COI gene fragment was amplified with universal COI primers: LCO1490 (5’-GGTCAACAAATCATAAAGATATTGG-3’) and HCO2198 (5’-TAAACTTCAGGGTGACCAAAAAATCA-3’), using a high-fidelity DNA polymerase, KOD-Plus-ver.2 (TOYOBO, Tokyo, Japan) [24–26]. The PCR products were purified using a FastGeneGel/PCR Extraction kit (NIPPON Genetics, Tokyo, Japan) to remove excessive primers, and the sequences were directly determined with a forward primer, as described above.

### Phylogenetic analysis

The obtained sequences were aligned with CLUSTAL W software [27] and examined using the program MEGA (Molecular Evolutionary Genetics Analysis) version 6 [28]. Phylogenetic trees were constructed by the maximum likelihood (ML) method with the best ML model selected based on the lowest BIC score (Bayesian Information Criterion) in MEGA 6 [28]. Branch support for the ML tree was calculated using the bootstrapping method with 1,000 replicates [28]. Phylogenetic analysis of the *Leishmania cyt* b gene included sequences from *L*. *(L*.*) infantum* (GenBank accession number: AB095958), *L*. *(L*.*) donovani* (AB095957), *L*. *(L*.*) major* (AB095961), *L*. *(L*.*) tropica* (AB095960), *L*. *(L*.*) amazonensis* (AB095964), *L*. *(L*.*) mexicana* (AB095963), *L*. *(V*.*) panamensis* (AB095968), *L*. *(V*.*) guyanensis* (AB095969), *L*. *(V*.*) braziliensis* (AB095966), *L*. *(V*.*) peruviana* (AB433282), *L*. *(V*.*) lainsoni* (AB433280), *L*. *(V*.*) naiffi* (AB433279), and *L*. *(V*.*) shawi* (AB433281). Phylogenetic analysis of the sand fly COI gene included sequences of *Pi*. *verrucarum* from the Department of Amazonas [Ama10 (FJ437242), Ama11 (FJ437243), and Ama14 (FJ437244)], Piura [Piu01 (AB984460) and Piu17 (FJ437264)], Cajamarca [Caj09 (FJ437247), Yuram02 (FJ437269), and Yumpe15 (FJ437267)], and Lima [Lim01 (AB984463) and Lim07 (FJ437256)] in Peru, and sequences of *Pi*. *maranonensis* from the Department of Cajamarca in Peru [Caja01 (LC593648), Caja02 (LC593649), Caja03 (LC593650), Caja04 (LC593651), Caja05 (LC593652), Caja06: (LC593653), Caja07 (LC593654), Caja08 (LC593655), Caja09 (LC593656), Caja10 (LC593657), Caja1H (LC593671), Caja5C (LC593672), Cola-a (LC593658), Cola-b (LC593659), Cola02 (LC593660), Cola03 (LC593661)], and Province of Chimborazo in Ecuador [Huig8C (LC593670)].

## Results

### Detection and identification of flagellates in sand flies

In the present study, 422 female sand flies were dissected for identification at the species level, and four species were recognized. Among them, the most dominant species, *Pi*. *verrucarum* (320 flies), was captured in all localities, and the remaining three species, *Pi*. *maranonensis* (83 flies), *Pi*. *robusta* (13 flies), and *Lu*. *castanea* (6 flies), were captured only in Nuevo Churuja (Table 1). Interestingly, *Pi*. *verrucarum* showed variations in the morphology of spermathecae, regardless of the collection sites; Some were shrinking, some were round, and the others were elongated (S1 Fig).

**Table 1:** Identification of sand fly species and detection of flagellates within sand flies by microscopic examination.

Locality	District	Sand fly species	No. captured	No. infected
La Curtiembre	Santo Tomas	*Pi*. *verrucarum*	59	0
Yerbabuena	La Jalca	*Pi*. *verrucarum*	39	0
Magdalena	Magdalena	*Pi*. *verrucarum*	29	0
Tingo Bajo	Tingo	*Pi*. *verrucarum*	176	1
Nuevo Churuja	Churuja	*Pi*. *verrucarum*	17	0
		*Pi*. *maranonensis*	83	1
		*Pi*. *robusta*	13	0
		*Lu*. *castanea*	6	0

**Fig 2:**
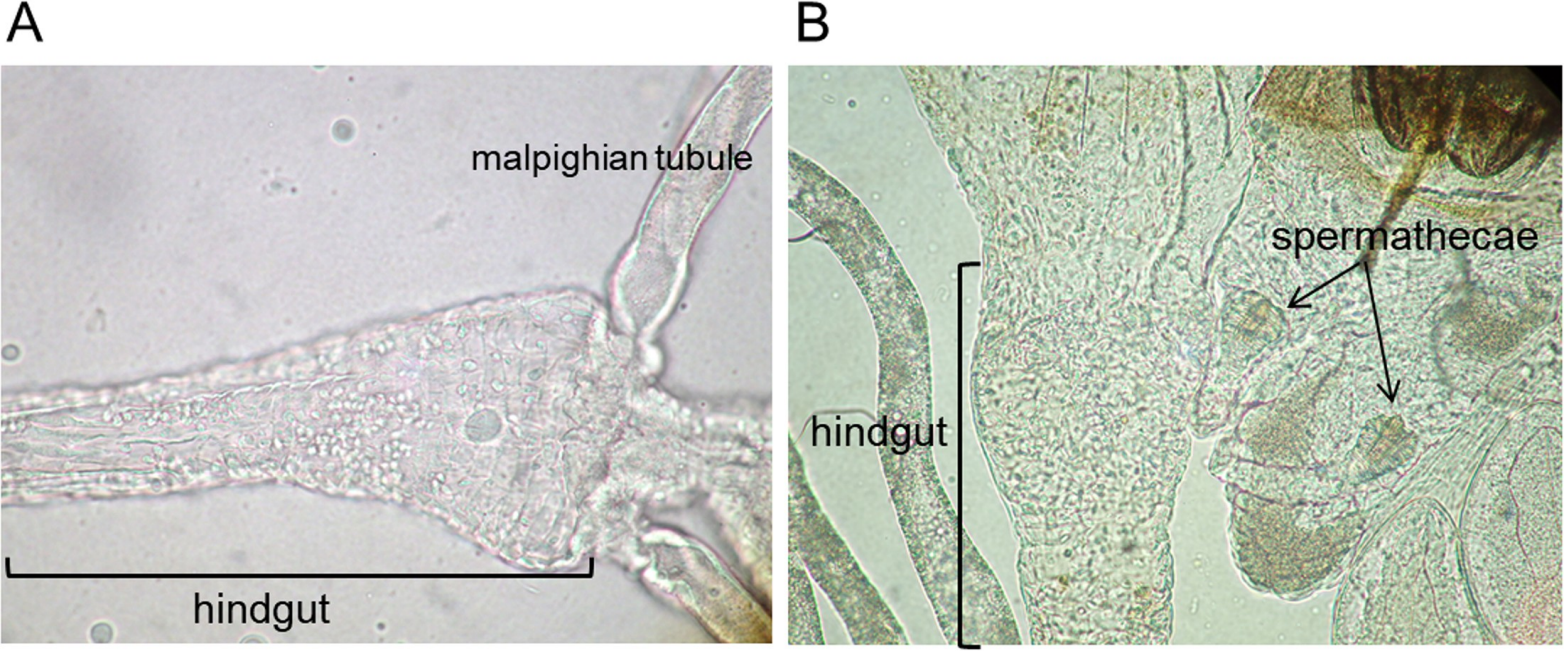
Natural infection of sand flies by flagellates. Natural infection of sand flies by flagellates was detected mainly in the hindgut of *Pi*. *verrucarum* from Tingo Bajo (A) and *Pi*. *maranonensis* from Nuevo Churuja (B).

**Fig 3:**
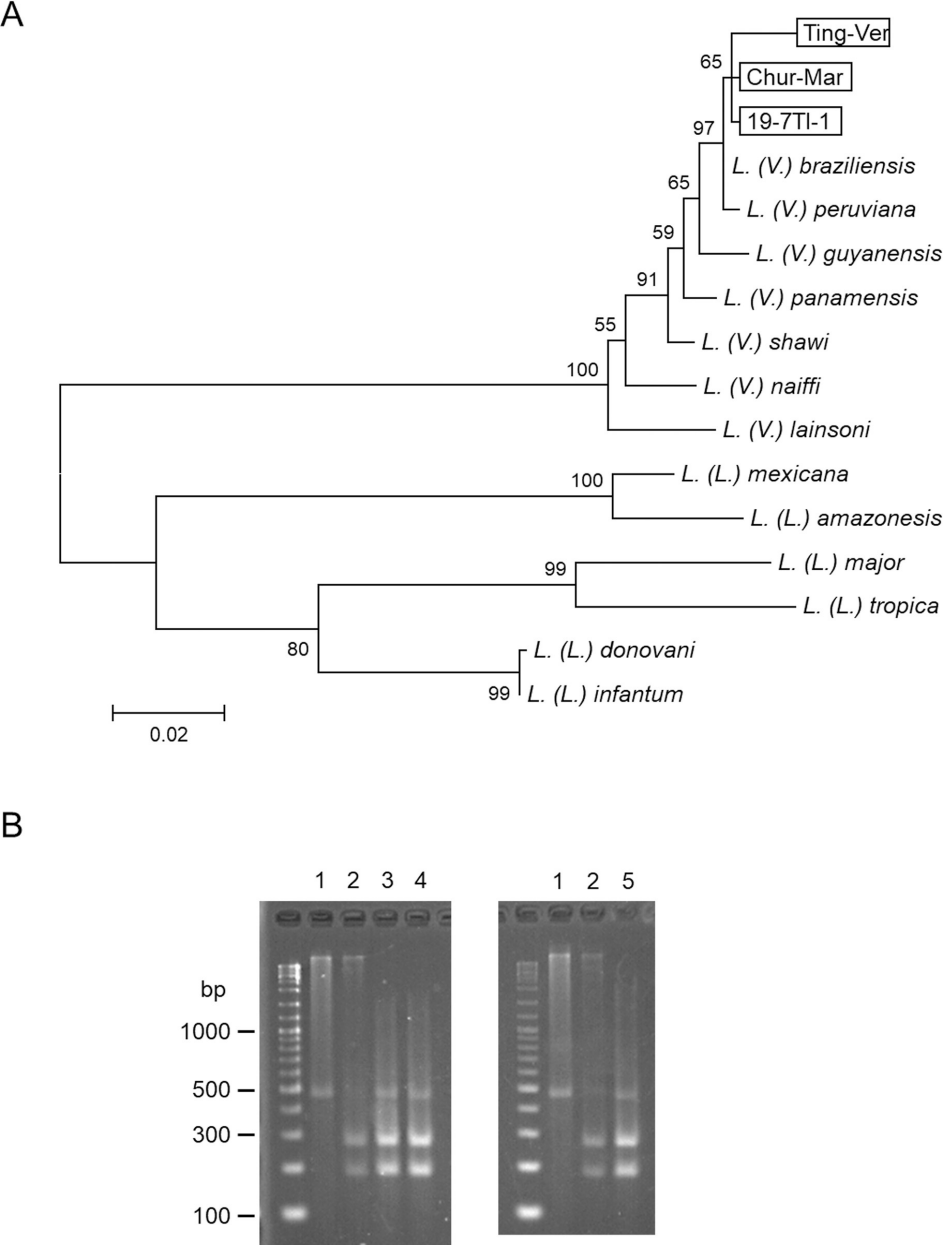
Identification of *Leishmania* species by cytochrome *b* and mannose phosphate isomerase gene analyses. A. A phylogenetic analysis of cytochrome *b* gene was performed by the maximum likelihood method together with sequences from 13 *Leishmania* species. The scale bar represents 0.02% divergence. Bootstrap values are shown above or below branches. Ting-Ver: *Leishmania*-positive *Pi*. *verrucarum* from Tingo Bajo, Chur-Mar: *Leishmania*-positive *Pi*. *maranonensis* from Nuevo Churuja, 19-7TI-1: a patient’s specimen from Tingo Bajo. B. PCR-RFLP analysis of mannose phosphate isomerase genes from *L*. *(V*.*) braziliensis* (lane 1), *L*. *(V*.*) peruviana* (lane 2), *Leishmania*-positive *Pi*. *verrucarum* from Tingo Bajo (lane 3), a patient’s specimen from Tingo Bajo (lane 4), and *Leishmania*-positive *Pi*. *maranonensis* from Nuevo Churuja (lane 5).

Natural infection of sand flies by flagellates was detected mainly in the hindgut of each one of *Pi*. *verrucarum* from Tingo Bajo and *Pi*. *maranonensis* from Nuevo Churuja under the microscope (Fig 2). The infection rates of *Pi*. *maranonensis* and *Pi*. *verrucarum* with *Leishmania* in the present study were 0.3% and 1.2%, respectively. The *cyt* b gene fragments were amplified from the parasites and subjected to sequence analyses. The *cyt* b gene sequences from *Pi*. *verrucarum* (Ting-Ver) (GenBank accession number: LC593674) and *Pi*. *maranonensis* (Chur-Mar) (LC593675) had a greater degree of homology with those of *L*. *(V*.*) peruviana* and *L*. *(V*.*) braziliensis* (98.9–99.7%) than other *Leishmania* species. A phylogenetic analysis showed that sequences from *Pi*. *verrucarum* (Ting-Ver) and *Pi*. *maranonensis* (Chur-Mar) could be divided into the clade of *L*. *(V*.*) peruviana* and *L*. *(V*.*) braziliensis* (Fig 3A). Parasite species from *Pi*. *verrucarum* (Ting-Ver) and *Pi*. *maranonensis* (Chur-Mar) were further differentiated by PCR-RFLP analysis of the *mpi* gene targeting a single-nucleotide polymorphism [7,8,16,17]. The RFLP pattern of both samples corresponded to that of *L*. *(V*.*) peruviana* but not *L*. *(V*.*) braziliensis* (Fig 3B), indicating that flagellates detected within *Pi*. *verrucarum* and *Pi*. *maranonensis* were both *L*. *(V*.*) peruviana*. This is the first report of the natural infection of *Pi*. *maranonensis* by *L*. *(V*.*) peruviana*.

### Identification of *Leishmania* species from a patient’s specimen

During this field research, a clinical sample was obtained from the cutaneous lesion of a patient with CL in Tingo Bajo where a flagellate-infected sand fly was detected. The *cyt* b gene fragment was analyzed, and the sequence (19-7TI-1) (GenBank accession number: LC593673) had a greater degree of homology with those of *L*. *(V*.*) peruviana* and *L*. *(V*.*) braziliensis* (99.5–99.7%) than others. The result was supported by a phylogenetic analysis (Fig 3A). PCR-RFLP analysis of the *mpi* gene showed that the parasite species was *L*. *(V*.*) peruviana* (Fig 3B).

### COI gene analyses of *Pintomyia verrucarum* and *Pintomyia maranonensis*

Since *Pi*. *verrucarum* showed morphological variations of spermathecae regardless of their habitat, genetic analysis based on the COI gene was performed on *Pi*. *verrucarum* and *Pi*. *maranonensis*, in which *Leishmania* infection was detected in this study, to identify their genetic diversities in comparison with those from other areas.

**Fig 4:**
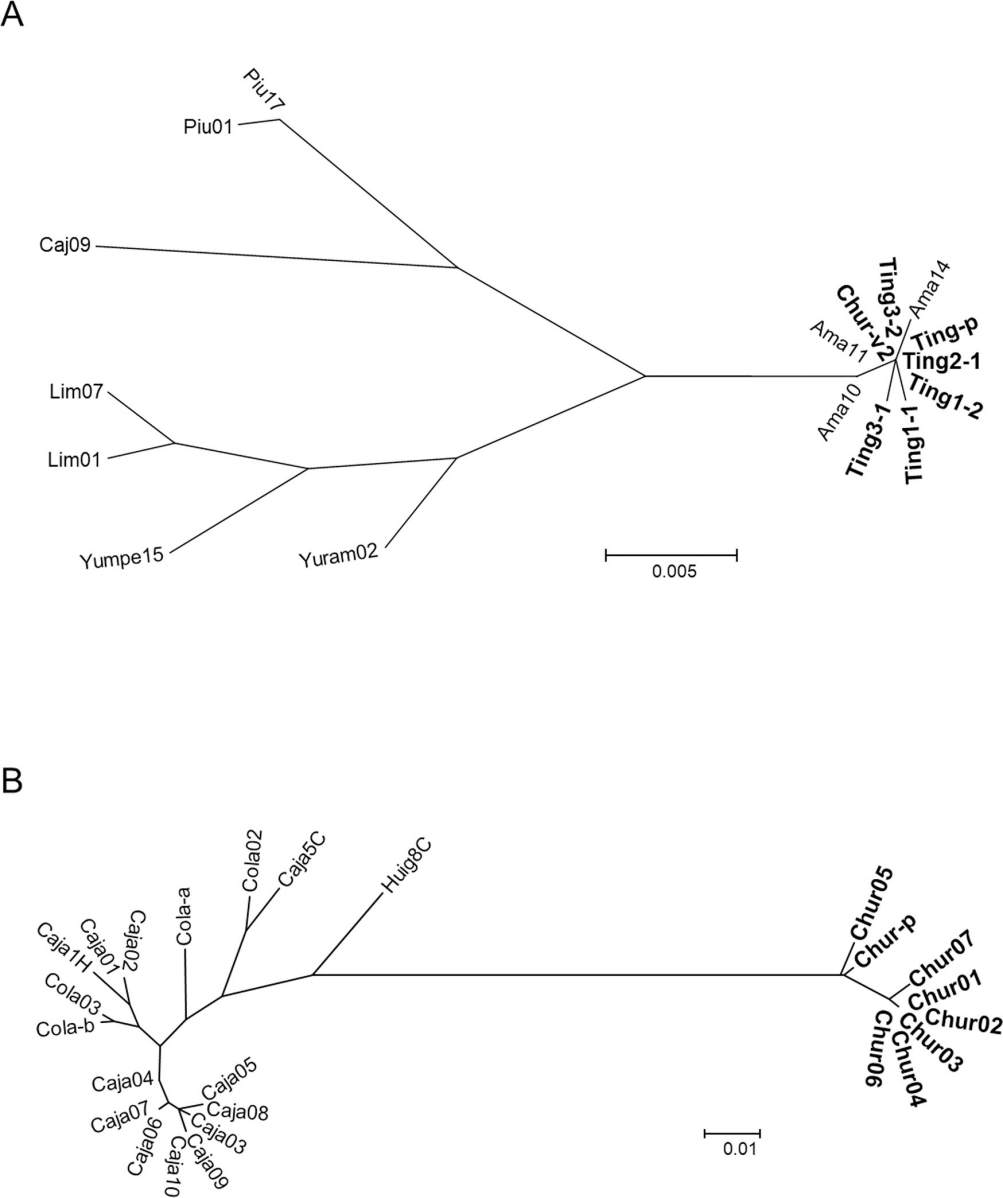
Phylogenetic analyses of cytochrome oxidase I (COI) gene sequences among *Pi*. *verrucarum* and among *Pi*. *maranonensis*. A. A phylogenetic analysis of the COI gene of *Pi*. *verrucarum* collected in this study (Ting1-1, Ting1-2, Ting2-1, Ting3-1, Ting3-2, Ting-p, and Chur-v2) was performed together with those from Departments of Amazonas (Ama10, Ama11, and Ama14), Piura (Piu01 and Piu17), Cajamarca (Caj09, Yuram02, and Yumpe15), and Lima (Lim01 and Lim07) in Peru. The scale bar represents 0.005% divergence. B. A phylogenetic analysis of the COI gene of *Pi*. *maranonensis* collected in this study (Chur01-Chur07 and Chur-p) was performed together with those from the Department of Cajamarca in Peru (Caja01-Caja10, Caja1H, Caja5C, Cola-a, Cola-b, Cola02, and Cola03) and the Province of Chimborazo in Ecuador (Huig8C). The scale bar represents 0.01% divergence.

Sequences of 630-bp COI gene fragments were determined in 7 *Pi*. *verrucarum* including the *Leishmania*-positive sample (Ting-p) collected in this study (GenBank accession numbers: LC593641- LC593647), and a phylogenetic analysis was performed together with those from other areas registered in GenBank (Fig 1). *Pintomyia verrucarum* captured in Tingo Bajo (Ting1-1, Ting1-2, Ting2-1, Ting3-1, Ting3-2, and Ting-p) and Nuevo Churuja (Chur-v2) of the Department of Amazonas were closely related to each other with genetic distances of 0.0–0.2% independent of the morphological variation of spermathecae (Fig 4A). COI gene sequences of *Pi*. *verrucarum* from the Department of Amazonas registered in GenBank (Ama10, Ama11, and Ama14) were located in the same clade as our samples. On the other hand, COI gene sequences of *Pi*. *verrucarum* from Departments of Piura (Piu01 and Piu17), Cajamarca (Caj09, Yuram02, and Yumpe15), and Lima (Lim01 and Lim07) composed different clades from those of Amazonas samples (Fig 4A). Genetic distances based on Kimura 2-parameter values were 2.4–2.8% between Amazonas and Piura samples, 2.1–3.1% between Amazonas and Cajamarca samples, and 2.3–2.8% between Amazonas and Lima samples, all of which were intraspecific diversity levels (<6.0%) [26].

COI gene sequences from 8 *Pi*. *maranonensis* including *Leishmania*-infected sample (Chur-p) collected in this study were also determined (GenBank accession numbers: LC593662- LC593669) and analyzed together with those from other areas in Peru and Ecuador (Fig 1). *Pintomyia maranonensis* captured in Nuevo Churuja (Chur01-Chur07 and Chur-p) were closely related to each other with genetic distances of 0.0–1.6% (Fig 4B). However, unexpectedly, they were distant from those obtained from other areas such as the Department of Cajamarca in Peru (Caja01-Caja10, Caja1H, Caja5C, Cola-a, Cola-b, Cola02, and Cola03), and the Province of Chimborazo in Ecuador (Huig8C), all of which are relatively closely related in spite of the geographic distance (Fig 4B). Genetic distances were 0.0–3.2% among Cajamarca samples and 3.9–4.1% between Cajamarca and Ecuador samples. On the other hand, genetic distances between Nuevo Churuja samples analyzed in this study and Cajamarca samples were 7.1–8.5%, which was over the intraspecific level (<6.0%) but mostly below the interspecific level (>8.4%) [26].

## Discussion

In the present study, the natural infection of sand flies by *Leishmania* was investigated in the Eastern Andes of northern Peru where cutaneous leishmaniasis caused by *L*. *(V*.*) peruviana* is endemic. Of 422 female sand flies examined, infection by flagellates was detected in *Pi*. *verrucarum* and *Pi*. *maranonensis*. Both flagellates were identified as *L*. *(V*.*) peruviana*, which is the causative agent of CL in these areas, strongly suggesting that *Pi*. *verrucarum* and *Pi*. *maranonensis* are responsible for the transmission of CL. This is the first report of the natural infection of *Pi*. *maranonensis* by *L*. *(V*.*) peruviana*. Further genetic analysis of these sand flies revealed that *Pi*. *verrucarum* was closely related to flies from other areas regardless of the morphological variation of spermathecae, whereas *Pi*. *maranonensis* was genetically unique when compared with flies from other areas.

The study area is located between the Central and Eastern Cordilleras of the Andes in northern Peru [29]. Epidemiological studies on leishmaniasis in the Peruvian Andes, including sand fly research, have been carried out mainly in endemic areas located in the North, Center, and South of the Western Andes, and occasionally in the North and Central inter-Andean valleys of the Central Andes and in the southern zone of the Eastern Andes. *Lu*. *ayacuchensis* and *Lu*. *peruensis*, which distribute in western valleys of the Central Andes and Northern and Central Andes, respectively, were identified as vectors of *L*. *(V*.*) peruviana*, the primary etiological agent of CL in the Peruvian Andes [13–16]. On the other hand, the vector responsible for the transmission of CL remains to be elucidated in endemic areas of the Eastern Andes. Interestingly, the distribution of both *Lu*. *ayacuchensis* and *Lu*. *peruensis* has not been reported in these areas, suggesting that some factors that may affect distributing sand fly species, such as the ecosystem, fauna, and flora, are unique in these areas.

In the study areas, *L*. *(V*.*) peruviana* was identified as the causative agent of CL in previous studies [7,8], and this was confirmed in the present study. The vector species responsible for the transmission of *L*. *(V*.*) peruviana* has not been identified. The present study detected flagellate parasites mainly in the hindguts of *Pi*. *verrucarum* and *Pi*. *maranonensis*, and both parasites were identified as *L*. *(V*.*) peruviana*, strongly suggesting that these sand fly species are responsible for the transmission of CL in these areas. *Pi*. *verrucarum* is widely distributed in Andean mountainous areas of the Eastern and Central Cordillera, and well-known as the primary vector of *Bartonella bacilliformis*, the etiologic agent of Carrion’s disease, also known as Oroya fever [12]. *Pintomyia verrucarum* was reported to have the capacity to transmit *L*. *(V*.*) peruviana* under experimental conditions [18], and the natural infection of the sand fly by unidentified *Leishmania* species was detected by PCR in an endemic area [19]. These findings strongly suggest that *Pi*. *verrucarum* is responsible for the transmission of *L*. *(V*.*) peruviana*. The present study showed that *Pi*. *verrucarum* is the natural vector of *L*. *(V*.*) peruviana* in endemic areas of CL for the first time. Moreover, natural infection by *L*. *(V*.*) peruviana* was detected unexpectedly in another sand fly species, *Pi*. *maranonensis*, suggesting that, at least, two sand fly species are associated with the transmission of *L*. *(V*.*) peruviana* in the study areas.

Since morphological variation of spermathecae was noted in *Pi*. *verrucarum* during the microscopic examination, genetic analysis of these flies was performed to identify the difference depending on the morphological characteristics. Interestingly, individuals of *Pi*. *verrucarum* in the study areas were closely related to each other regardless of the morphological variation, and their genetic diversities were within the intraspecific level when compared with those from other areas, suggesting that spermathecae of *Pi*. *verrucarum* are more flexible than those of other species. This finding should be taken into account on the morphological identification of sand flies, especially when unfixed specimens are used. Another *Leishmania*-positive sand fly species, *Pi*. *maranonensis*, was also subjected to genetic analysis, although morphological variation was not observed. Unexpectedly, *Pi*. *maranonensis* in the study area was genetically distant from flies of other areas with genetic distances between intraspecific and interspecific levels. This is the first report on the transmission of *Leishmania* species by *Pi*. *maranonensis*. Since *Pi*. *maranonensis* in the study area may have unique characteristics of the intestinal environment that result in acquiring the vectorial capacity, further studies associated with vector competence, such as mid- and hindgut molecules and the microbiome, would be interesting [30–32].

In the present study, the natural infection of sand flies by *Leishmania* was microscopically examined in the northern Peruvian Andes on the Cordillera Central where CL caused by *L*. *(V*.*) peruviana* is endemic. Both flagellates detected within *Pi*. *verrucarum* and *Pi*. *maranonensis* were identified as *L*. *(V*.*) peruviana*, strongly suggesting that *Pi*. *verrucarum* and *Pi*. *maranonensis* are responsible for the transmission of leishmaniasis in these areas. Genetic divergence of *Pi*. *verrucarum* in these areas was confirmed to be at the intraspecific level regardless of their morphological variations, whereas *Pi*. *maranonensis* was genetically characteristic regarding the genetic distance between intra- and interspecific levels when compared with flies from other areas. Since *Pi*. *maranonensis* has never reported to transmit *Leishmania* species, this species in the study area is expected to have unique characteristics associated with the vectorial capacity. Further studies on the microenvironment of the mid- and hindgut, in which particular parasite species can develop, will be expected to provide further insight into the vector competence of sand flies. On the other hand, more detailed morphological and molecular analyses of *Pi*. *maranonensis* in the study area will be needed since the possibility of a new species cannot be ruled out, based on the finding that genetic distances of the COI gene with those from other areas were over the intraspecific level.

## Supporting information

S1 Fig Morphological variations of spermathecae among *Pifanomyia verrucarum*. (TIF)
