# Supplementary figures and images for: Natural infections of Pintomyia verrucarum and Pintomyia maranonensis by Leishmania (Viannia) peruviana in the Eastern Andes of northern Peru
Source: PLoS Negl Trop Dis. 2021 Apr 15;15(4):e0009352. doi: 10.1371/journal.pntd.0009352 (PMC8078796; doi:10.1371/journal.pntd.0009352)

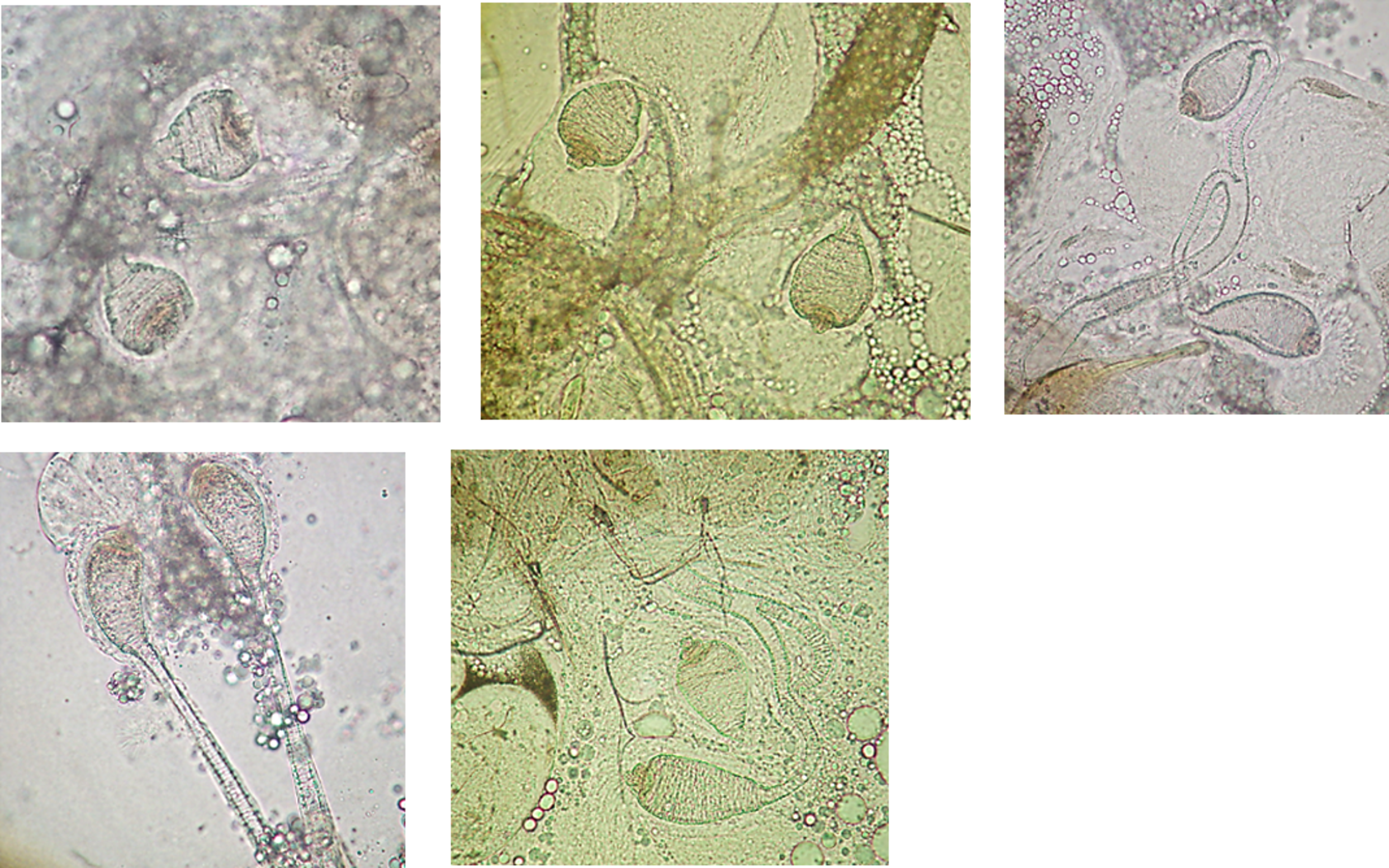

Supplement: S1 Fig — (TIF) [file pntd.0009352.s001.tif]
